# Quantitative bone SPECT/CT-derived tumor burden as a prognostic biomarker in metastatic castration-resistant prostate cancer treated with Ra-223

**DOI:** 10.1186/s41824-026-00309-4

**Published:** 2026-07-13

**Authors:** Yoshimitsu Fukushima, Go Kimura, Jun Akatsuka, Yoshimitsu Honda, Yujiro Nakaoka, Hiromitsu Hayashi, Shinichiro Kumita

**Affiliations:** 1 Department of Radiology, Nippon Medical School, 1-1-5 Sendagi, Bunkyo-ku, Tokyo, 113-8603 Japan; 2 Department of Urology, Nippon Medical School, 1-1-5 Sendagi, Bunkyo-ku, Tokyo, 113-8603 Japan

**Keywords:** Bone SPECT/CT, Castration-resistant prostate cancer, Ra-223, Bone metastases, Prognostic biomarker

## Abstract

**Purpose:**

Metastatic castration-resistant prostate cancer (mCRPC) is bone-predominant, requiring objective imaging biomarkers to guide Ra-223 therapy. We evaluated overall metabolic bone volume (OMBV) and overall total bone uptake (OTBU) from bone SPECT/CT as prognostic markers in mCRPC patients treated with Ra-223.

**Methods:**

Thirty mCRPC patients underwent pre-treatment planar and SPECT/CT bone scintigraphy, with OMBV and OTBU derived using GI-BONE software. Overall survival (OS) was the primary endpoint; progression-free survival (PFS) and time to symptomatic skeletal event (TTSE) were secondary. Receiver operating characteristic analysis (Youden index) defined cut-offs for OS and TTSE independently. Treatment response was classified by composite changes in OMBV, OTBU, and bone scan index. OS, OMBV, and OTBU were analyzed by univariate Cox regression as continuous and dichotomized variables.

**Results:**

Baseline skeletal burden was substantial (median OMBV 1,191.21 cm^3^ [IQR 93.80–1,948.01]; median OTBU 9,746.18 [1,280.01– 17,587.11]); 87% had extent of disease grade 3–4. Post-treatment imaging in 24 patients yielded 10 responders (42%) and 14 non-responders (58%), with responder mean OMBV and OTBU reductions of 57% and 68%. Dichotomized baseline OMBV (hazard ratio [HR] 6.0320, 95% confidence interval [CI] 1.6880–21.5546; *p* < 0.001) and OTBU (HR 9.4492, 95% CI 2.0968–42.5826; *p* < 0.001) were significant predictors of OS. OMBV and OTBU were not significantly associated with PFS or TTSE.

**Conclusions:**

OMBV and OTBU from bone SPECT/CT may provide useful prognostic information in mCRPC patients undergoing Ra-223 therapy, offering objective quantification of skeletal tumor burden using widely available technology. Further validation in larger cohorts is warranted.

## Introduction

Castration-resistant prostate cancer (CRPC) is defined as disease progression despite castrate levels of testosterone (serum testosterone < 50 ng/dL or 1.7 nmol/L), manifested by rising prostate-specific antigen (PSA) or radiographic progression. According to the Prostate Cancer Working Group 3 (PCWG3) criteria, a minimum starting PSA value of 1.0 ng/mL is required when PSA elevation is the sole evidence of progression (Scher et al. 2016; Lange and Vessella 1998; Bubendorf et al. 2000). Metastatic CRPC (mCRPC) is a bone-predominant disease, with approximately 90% of patients affected by skeletal lesions. The axial skeleton, which contains the principal sites of active bone marrow, is the most common site of metastatic prostate cancer (Saad et al. 2007; Roodman 2004). Bone lesions in mCRPC are typically characterized as osteoblastic or mixed on imaging, and bone-related parameters have been established as significant prognostic factors for overall survival (OS) in these patients (Cook et al. 2006; Fizazi et al. 2015). Bone metastases cause serious complications, including pain, pathological fractures, and neurological dysfunction, significantly impairing patients’ quality of life (QOL) (Yap et al. 2003; Weinfurt et al. 2005). Depletion of hematopoietic bone marrow by tumor expansion has been recognized as a significant cause of cancer-specific death in mCRPC (Logothetis and Lin 2005). Skeletal-related events (SREs), including radiation therapy to bone, pathological fractures, spinal cord compression, and bone surgery, result in clinically meaningful deterioration in health-related QOL, affecting physical, functional, and emotional well-being (Weinfurt et al. 2004; Patrick et al. 2015). These findings underscore the importance of accurately assessing bone metastatic burden for risk stratification and treatment planning.

Prognostic stratification in mCRPC is essential for treatment planning and clinical trial design. Several clinical and biochemical parameters have been established as prognostic factors, including PSA doubling time (PSADT), performance status, alkaline phosphatase (ALP), lactate dehydrogenase (LDH), hemoglobin, and presence of visceral metastases (Halabi et al. 2014; Armstrong et al. 2010). Bone-related parameters are influential prognostic factors for OS in patients with skeletal involvement. Low baseline ALP (≤143 U/L), bone-specific alkaline phosphatase (BSAP) <146 U/L, corrected urinary N-telopeptide (uNTx) ≤50 nmol/mmol, and absence of prior SREs have been associated with improved prognosis (Saad et al. 2004; Brown et al. 2005).

The therapeutic landscape for mCRPC has evolved substantially over the past decade. Bisphosphonates, such as zoledronate, and molecularly targeted agents, such as denosumab, are commonly used to delay progression of bone metastasis and reduce the risk of SREs (Saad et al. 2007; Fizazi et al. 2011). Androgen receptor signaling inhibitors (ARSIs), including abiraterone and enzalutamide, have demonstrated overall survival benefits in both chemotherapy-naive and post-docetaxel settings (de Bono Js et al. 2011; Ryan et al. 2013; Scher et al. 2012). Taxane chemotherapy with docetaxel and cabazitaxel remains a cornerstone of mCRPC treatment. Among bone-targeting radiopharmaceuticals, beta-emitting agents such as Sr-89 provide pain palliation but do not improve survival (Bodei et al. 2008). Ra-223, an alpha-emitting radiopharmaceutical, is the only bone-targeting radionuclide that has demonstrated OS improvement in symptomatic mCRPC patients. The ALSYMPCA trial demonstrated prolonged overall survival (median: 14.9 vs 11.3 months; hazard ratio [HR]: 0.70; *p* < 0.001) and delayed symptomatic skeletal events (median: 15.6 vs 9.8 months; HR: 0.66), with minimal myelosuppression (Bruland et al. 2006; Parker et al. 2013). ^177^Lu-PSMA-617 has also emerged as an effective radioligand therapy for patients with PSMA-positive mCRPC (Sartor et al. 2021). Accurate assessment of baseline bone metastatic burden is important for patient selection and prognostic stratification.

Bone scintigraphy using ^99m^Tc-labeled diphosphonates remains the standard imaging modality for detecting bone metastases in mCRPC. However, conventional planar bone scintigraphy has inherent limitations: it does not explicitly identify cancer, may paradoxically worsen during treatment response (flare phenomenon), and provides incomplete quantification of disease extent (Rybak and Rosenthal 2001; Cook et al. 2016). The extent of disease (EOD) scale and bone scan index (BSI) have been developed to assess metastatic burden semi-quantitatively (Soloway et al. 1988; Jorgensen et al. 1995; Imbriaco et al. 1998; Ulmert et al. 2012; Dennis et al. 2012). BSI quantifies total skeletal tumor burden as a percentage of total skeletal mass and demonstrates high intra- and inter-reviewer reproducibility (correlation coefficients 0.97–0.94). Computer-aided diagnosis systems such as BONENAVI (FUJIFILM PDRadiopharma, Tokyo, Japan) enable automated BSI quantification with 100% reproducibility, and on-treatment changes in BSI serve as indicators of treatment response in mCRPC (Kaboteh et al. 2013; Miyoshi et al. 2016). The introduction of ^99m^Tc-labeled diphosphonate single photon emission computed tomography/computed tomography (SPECT/CT) has substantially improved sensitivity and diagnostic accuracy compared with planar bone scintigraphy. SPECT/CT provides higher diagnostic confidence and inter-reviewer agreement than bone scintigraphy alone (Utsunomiya et al. 2006). ^18^F-sodium fluoride (NaF) positron emission tomography/computed tomography (PET/CT) offers superior image resolution and absolute quantification, demonstrating higher sensitivity and specificity for detecting bone metastases in high-risk prostate cancer patients than bone scintigraphy and ^18^F-FDG PET/CT (Even-Sapir et al. 2006). However, NaF-PET/CT remains rarely available in Japan, making quantitative bone SPECT/CT an attractive alternative. Recent advances in quantitative SPECT/CT have enabled objective measurement of bone metastatic burden through volumetric parameters. Novel indices, including lesion uptake volume (LUV) and total lesion uptake (TLU), have been proposed as three-dimensional quantitative markers derived from ^99m^Tc-diphosphonate SPECT/CT. LUV represents the extracted volume of bone metastases showing high uptake above a standardized uptake value (SUV) threshold, while TLU is the product of mean SUV and LUV. These volumetric parameters provide higher prognostic value than conventional EOD or BSI and serve as independent prognostic factors for cancer-specific survival in mCRPC patients (Umeda et al. 2018). SUV values obtained from quantitative SPECT/CT can discriminate bone metastases from degenerative changes, functioning as an osteoblastic biomarker. Similarly, ^18^F-NaF PET/CT-derived indices such as total fluoride skeletal metastatic lesion uptake (TLF10) and total volume of fluoride-avid bone metastases (FTV10) have been established as significant independent predictors of OS and SRE risk (Etchebehere et al. 2015).

This study used bone SPECT/CT and GI-BONE quantification software (Nihon Medi-Physics Co., Ltd., Tokyo, Japan) to examine patients with CRPC and bone metastases undergoing Ra-223 therapy. The objectives were to assess the severity and extent of bone metastases using volumetric parameters (OMBV and OTBU), to evaluate patient prognoses, and to determine the prognostic value of these quantitative indices. We hypothesized that pre-treatment OMBV and OTBU may help stratify patient prognosis and complement established clinical and biochemical prognostic markers.

## Materials and methods

### Patient population

**Fig. 1:**
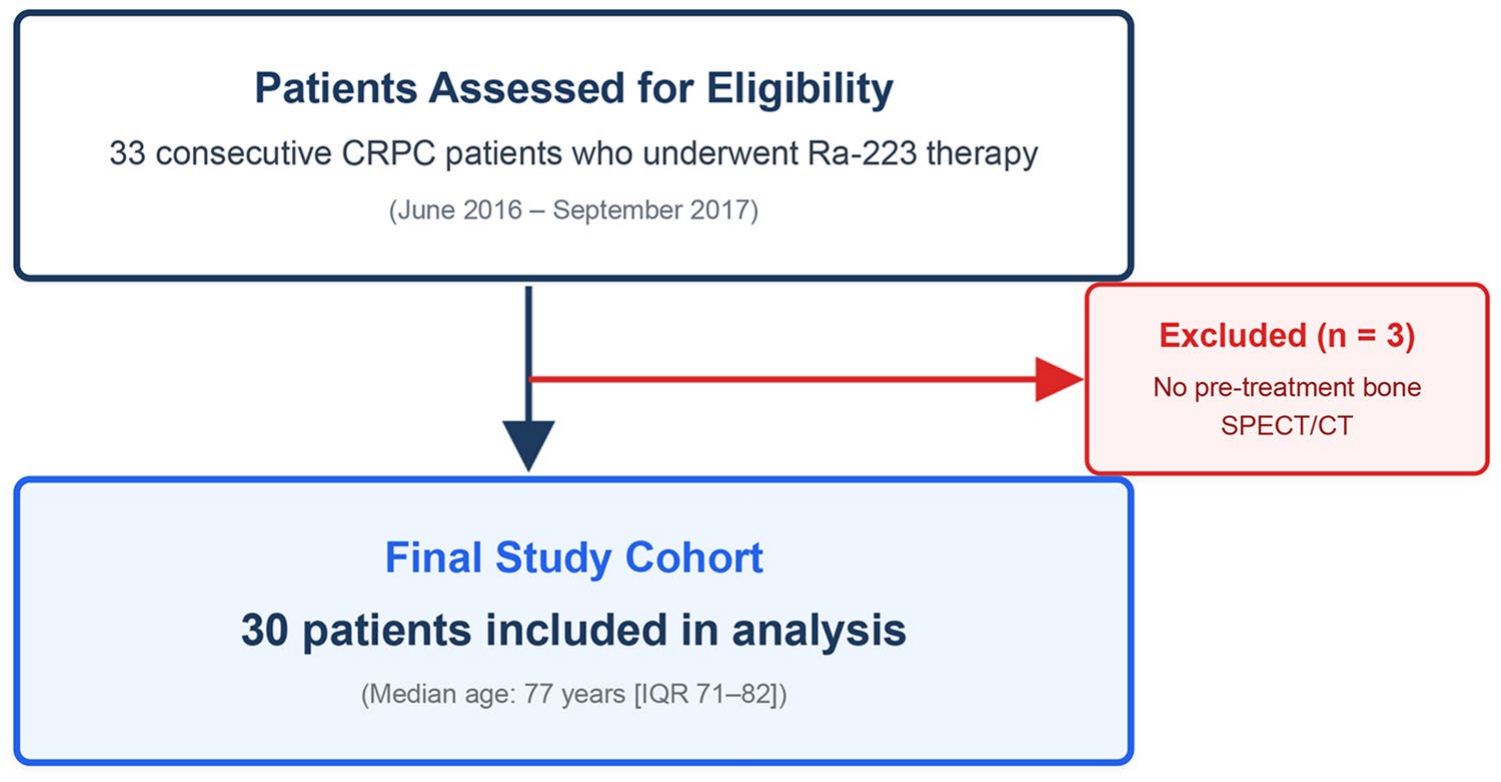
Flowchart of patients included and excluded in this study

This retrospective study examined 33 consecutive patients with CRPC (median age 74 years [IQR 68–80]) who underwent Ra-223 therapy at our institution between June 2016 and September 2017, with a 5-year follow-up. All patients had histologically confirmed prostate adenocarcinoma with documented progression to castration-resistant status, defined as disease progression despite serum testosterone levels below 50 ng/dL achieved through surgical or medical castration. Inclusion criteria required the presence of symptomatic bone metastases, Eastern Cooperative Oncology Group (ECOG) performance status of 0–2, adequate hematologic function (absolute neutrophil count ≥ 1.5 × 10^9^/L, platelet count ≥ 100 × 10^9^/L, hemoglobin ≥ 10 g/dL), and no known visceral metastases. These patients presented with bone metastases and, occasionally, slight lymph node metastases. Clinical diagnoses were confirmed based on prostate biopsies, serum PSA tests, and bone SPECT/CT findings. Baseline laboratory assessments included PSA, LDH, ALP, bone-specific alkaline phosphatase (BAP), and type I collagen telopeptide (ICTP). Three patients who did not undergo pre-treatment bone SPECT/CT were excluded, yielding a final study cohort of 30 patients (77 [71–82] years old) (Fig. 1). The study protocol was approved by the Institutional Review Board of Nippon Medical School (approval number: 30–12-1051), and the requirement for informed consent was waived due to the retrospective nature of the study, in accordance with the institutional guidelines for retrospective observational research with information disclosure procedure (opt-out).

### Bone scintigraphy and SPECT/CT imaging

All patients underwent pre-treatment bone scintigraphy within 4 weeks before initiating Ra-223 therapy. Patients were instructed to maintain adequate hydration and void immediately before imaging to minimize bladder activity artifact. All imaging was performed on a Symbia T2 SPECT/CT system (Siemens Healthineers, Erlangen, Germany), a dual-head gamma camera–based device equipped with low-energy high-resolution collimators. Planar whole-body images (anterior and posterior views, from vertex to toes) were acquired 2–3 hours after intravenous administration of 740 MBq of technetium-99 m diphosphonates at a scanning speed of 15 cm/min. Subsequently, SPECT and low-dose CT images were acquired on the same system. To balance comprehensive disease assessment with acquisition time, an uptake-driven adaptive SPECT/CT protocol was applied: whole-body planar scintigraphy was reviewed first, and SPECT/CT bed positions were then tailored to cover all skeletal regions demonstrating focal tracer uptake on planar imaging. Accordingly, patients without skull uptake on planar images were scanned with 2 bed positions (shoulder to upper thigh), while patients with skull uptake were scanned with 3 bed positions (vertex to upper thigh), ensuring that every region of suspected metastatic involvement was covered by SPECT/CT. SPECT images were acquired over 5–7 minutes per bed position using a 128 × 128 matrix with 60 projections over 360 degrees. Images were reconstructed using the Flash3D iterative algorithm with 6 subsets and 8 iterations, incorporating CT-based attenuation correction and scatter correction. Low-dose CT (110 kV, 10–40 mAs) was performed for anatomical localization and attenuation correction purposes.

### Quantitative analysis using GI-BONE

Quantitative analysis was performed using GI-BONE, a dedicated bone SPECT/CT analysis tool that enables SUV quantification and volumetric assessment of skeletal lesions, which has been applied in prior quantitative bone SPECT/CT studies, including methodological evaluations of test–retest reproducibility and disease-specific applications (Yamane et al. 2021; Moridera et al. 2022). The software automatically segments skeletal structures using CT-based anatomical boundaries and calculates metabolic bone volume (MBV) and total bone uptake (TBU) for each identified lesion. SUV was calculated using the standard formula: SUV = tissue activity concentration (Bq/mL)/(injected dose [Bq]/body weight [g]). Hot spots with SUV ≥ 4.50 were automatically identified as volumes of interest. The rationale for this threshold was grounded in reference values for normal skeletal uptake on ^99m^Tc-diphosphonate SPECT/CT. Umeda et al. previously proposed a threshold of SUV 7.00 based on discrimination between metastatic and non-metastatic skeletal regions in a mixed cohort (Umeda et al. 2018). In the present study, we selected a lower threshold of SUV 4.50 to more fully capture osteoblastic activity along the spectrum from physiologic background to active metastasis; this value approximates the population mean of normal ^99m^Tc-diphosphonate skeletal uptake reported in large-scale reference studies (mean spine/pelvis SUVbw 4.57 ± 1.97 (Hou et al. 2024); normal vertebral SUVmax 6.5–8.1 (Kuji et al. 2017; Rohani et al. 2020). OTBU was therefore defined as total skeletal activity at or above the physiologic uptake floor, encompassing both background osteoblastic activity and pathologic metastatic uptake. Two experienced nuclear medicine physicians (with 29 and 10 years of experience in bone scintigraphy interpretation, respectively) independently reviewed all images. They reached a consensus on lesion identification through joint discussion when discrepancies arose. Benign lesions (such as degenerative joint disease, fracture healing, and Paget’s disease) and physiological accumulations (such as kidney, bladder, and injection site activity) were manually excluded based on CT morphology and clinical information, leaving only active bone metastases for analysis. For each metastatic lesion, MBV (cm^3^) was defined as the volume of voxels exceeding the SUV threshold, and TBU was calculated as the product of MBV and mean SUV within the lesion volume. OMBV was calculated as the sum of all individual lesion MBVs, representing the total volume of metabolically active bone metastases throughout the skeleton. OTBU was calculated as the sum of all individual lesion TBUs, representing the cumulative metabolic activity across all skeletal metastases. EOD grade (classified as 0–4 based on the number and distribution of lesions) and BSI (expressed as the percentage of total skeletal mass involved by tumor) were calculated using BONENAVI applied to planar bone scintigraphy images for comparison with the SPECT/CT-derived volumetric parameters. Receiver operating characteristic (ROC) analyses were performed to determine optimal cut-off values for OMBV and OTBU for prognostic stratification, using overall survival status at 2 years as the classification endpoint. The optimal cut-off was determined using the Youden index (sensitivity + specificity − 1).

### Ra-223 therapy and follow-up

All patients received Ra-223 dichloride (Xofigo, Bayer HealthCare Pharmaceuticals Inc., Berlin, Germany) therapy after completion of pre-treatment imaging assessments. Ra-223 was administered intravenously at a dose of 55 kBq/kg body weight every 4 weeks for up to 6 cycles, according to the approved dosing regimen established in the ALSYMPCA trial. Patients were monitored for hematologic toxicity with complete blood counts performed before each injection cycle. Dose modifications or treatment discontinuation were implemented in accordance with institutional guidelines for significant cytopenias or disease progression. Concomitant medications, including bone-modifying agents (BMAs; bisphosphonates or denosumab), ARSIs, and corticosteroids, were continued as clinically indicated. Prior systemic therapies for CRPC were enumerated using an operational rule informed by PCWG3 recommendations (Scher et al. 2016): each AR-targeted agent (abiraterone, enzalutamide), chemotherapy (docetaxel, cabazitaxel, carboplatin, epirubicin, etoposide), estramustine (counted as chemotherapy), and secondary hormonal manipulation (ethinylestradiol, and antiandrogen switch or withdrawal operationally defined as use of both bicalutamide and flutamide in the castration-resistant phase) administered before Ra-223 was counted as one line. Continuous androgen-deprivation therapy and the initial combined androgen blockade during the castration-sensitive phase were not counted. Patients were dichotomized at ≥ 3 versus <3 prior lines, a threshold chosen a priori as a clinically meaningful separation that is consistent with the observed median prior-line count in our cohort. Therapeutic efficacy was evaluated approximately 1 month after the final Ra-223 injection using repeat bone SPECT/CT with the identical imaging protocol. Treatment response was classified through a composite assessment integrating serial quantitative bone SPECT/CT and planar imaging parameters. Patients were designated as responders when they demonstrated either (i) concurrent decrease in both OMBV and OTBU on post-treatment SPECT/CT, or (ii) major decrease (≥50%) in BSI accompanied by stable or declining SPECT/CT volumetric parameters. Non-responders were defined as patients failing both criteria, manifested by increases in OMBV, OTBU, or overall skeletal tumor burden on post-treatment imaging. Comparisons of pre- and post-treatment OMBV, OTBU, and other imaging parameters were then performed within each response category. Patients were followed prospectively for up to 5 years after treatment initiation. The primary endpoint was OS, defined as the time from first Ra-223 injection to death from any cause. Secondary endpoints were progression-free survival (PFS), defined as the time from first Ra-223 injection to the first imaging-confirmed progression on bone SPECT/CT, whole-body bone scintigraphy, or cross-sectional imaging (aligning with the PCWG3 radiographic PFS philosophy, with PSA-only progression excluded because the reliability of bone-turnover serum markers was limited in this cohort); and time to first symptomatic skeletal event (TTSE), defined as the time to occurrence of pathological fracture, spinal cord compression, radiation therapy to bone, or bone surgery. PFS events were ascertained from each patient’s institutional imaging archive and clinical progression notes, with the progression date confirmed by both the nuclear medicine investigator and the managing urologist. For patients who transferred to another institution during follow-up, progression events were obtained from transfer records and subsequent correspondence, and the date of the first documented imaging-confirmed progression was used. The median follow-up of 12 months reflects the interval from Ra-223 initiation to the administrative censoring date for OS and TTSE, whereas PFS captured the shorter time to the first imaging-confirmed progression within that window; accordingly, all 30 patients had a documented progression event before OS censoring, and no PFS observations were censored.

### Statistical analyses

Statistical analyses were performed to evaluate the prognostic value of quantitative SPECT/CT-derived parameters. Sample size was determined based on previous studies evaluating imaging biomarkers in mCRPC, with a minimum of 30 patients considered adequate for exploratory prognostic analyses. Continuous variables were expressed as medians with interquartile ranges (IQR), and categorical variables were expressed as frequencies and percentages. The Shapiro-Wilk test was used to assess the normality of continuous variables. Pre- and post-treatment values were compared using the Wilcoxon signed-rank test for paired samples, given the non-normal distribution of imaging parameters. Comparisons between responders and non-responders were performed using the Mann-Whitney U test for continuous variables and Fisher’s exact test for categorical variables. Event-free survival curves were estimated using the Kaplan-Meier method, and differences between groups were assessed using the log-rank test. For OS and TTSE, patients were stratified into low and high groups based on independent ROC-derived optimal cut-off values for OMBV and OTBU, using the Youden index applied to 2-year OS event status and 2-year symptomatic skeletal event status, respectively, as classification endpoints. The resulting cut-offs were OMBV 412.74 cm^3^ and OTBU 2,592.59 for OS, and OMBV 417.22 cm^3^ and OTBU 2,592.59 for TTSE. For PFS, OMBV and OTBU were analyzed as continuous variables. Univariate Cox proportional hazards regression was performed to evaluate factors associated with OS, PFS, and TTSE, with results expressed as hazard ratios (HRs) and 95% confidence intervals (CIs). The proportional hazards assumption was verified by examining Schoenfeld residuals, and no variable reported in Tables 4–6 violated the assumption. Variables with *p* < 0.05 in univariate analysis were considered statistically significant. Because the univariate analyses included 20 candidate variables evaluated across three endpoints (60 tests in total), these *p* values were not adjusted for multiple comparisons; under a Bonferroni-corrected family-wise threshold of 0.05/60 ≈ 0.00083, OMBV, OTBU, and completion of the full Ra-223 course for OS would retain significance, whereas borderline associations (*p* ≈ 0.03–0.05) should be interpreted as hypothesis-generating. Multivariate analysis was not performed due to the limited sample size relative to the number of candidate variables, in line with the recommendation of approximately 10 events per variable to avoid overfitting. All statistical tests were two-sided, and *p* < 0.05 was considered statistically significant. All statistical analyses were performed with BellCurve for Excel (Social Survey Research Information, Tokyo, Japan).

## Results

Overall, 30 patients (median age 77 years [IQR 71–82]) underwent pre-treatment bone SPECT/CT and Ra-223 therapy. The majority of patients had high-grade disease (Gleason score 8–10: 83%) with extensive skeletal involvement (EOD grade 3–4: 87%). Baseline clinical characteristics and laboratory parameters are summarized in Table 1. The median baseline OMBV was 708.35 cm^3^ (IQR 330.20–1,466.48), and the median OTBU was 5,908.59 (IQR 2,268.89–13,873.64), reflecting substantial heterogeneity in skeletal tumor burden across the cohort. ROC analysis identified optimal cut-off values for prognostic stratification (OS: OMBV 412.74 cm^3^, OTBU 2,592.59; TTSE: OMBV 417.22 cm^3^, OTBU 2,592.59), and patients were divided into low and high groups accordingly.

**Table 1:** Patient characteristics

Parameter	Value
Number of patients	30
Age (years)	77 (71–82)
GS 6/7/8/9/10	1/4/5/20/0
T stage T1/T2/T3/T4	1/6/12/11
N stage N0/N1	20/10
M stage M0/M1a/M1b/M1c	4/0/20/6
Time to CRPC (months)	22.0 (13.0–59.5)
Number of CRPC treatment lines before Ra-223 per PCWG3 (0/1/2/3/≥4)	2/1/9/11/7
Prior CRPC treatment lines per PCWG3, median [IQR]	3 (Lange and Vessella 1998; Bubendorf et al. 2000)
**Tumor markers at Ra-223 therapy**	
PSA (ng/mL)	33.88 (8.71–92.68)
LDH (IU/L)	221 (198–304)
**Bone markers at Ra-223 therapy**	
ALP (IU/L)	313 (211–509)
BAP (IU/L)	27.1 (10.5–47.0)
ICTP (ng/mL)	7.1 (4.9–13.1)
**Imaging findings at Ra-223 therapy**	
EOD 1/2/3/4	2/2/14/12
Concomitant therapy during Ra-223	
Enzalutamide	12 (40%)
Abiraterone	12 (40%)
PSL	20 (67%)
BMA	19 (63%)
Non-concomitant ARSI exposure*	
Enzalutamide	13 (43%)
Abiraterone	10 (33%)
Any ARSI	6 (20%)

Note: Data are presented as median (IQR) for continuous variables and n (%) for categorical variables. GS = Gleason score; PSA = prostate-specific antigen; LDH = lactate dehydrogenase; ALP = alkaline phosphatase; BAP = bone-specific alkaline phosphatase; ICTP = type I collagen telopeptide; EOD = extent of disease; PSL = prednisolone; BMA = bone-modifying agent

Twenty-two of 30 patients (73%) completed the full 6-cycle Ra-223 course. Eight patients discontinued treatment early due to disease progression (*n* = 5), hematologic toxicity (*n* = 2), or patient preference (*n* = 1). Notably, patients with high baseline OTBU were significantly less likely to complete the full treatment course than those with low baseline OTBU (64% vs 100%, *p* = 0.045), suggesting that higher baseline tumor burden may be associated with treatment tolerability. Post-treatment bone SPECT/CT was performed in 24 patients approximately one month after the final injection. Treatment response was evaluable in these patients: 10 (42%) demonstrated a positive metabolic response, while 14 (58%) showed no response or disease progression. Among responders, OMBV and OTBU significantly decreased from baseline (*p* < 0.05), with reductions in median values of 57% and 68%, respectively, while conventional parameters, including EOD, BSI, and SUVmax, showed no significant changes (Table 2). In contrast, among non-responders, OMBV and OTBU significantly increased (*p* < 0.05), accompanied by increases in BSI and SUVmax (Table 3), indicating progressive skeletal disease despite treatment.

**Table 2:** Change in parameters between pre- and post-treatment among patients with positive responses to Ra-223 therapy

N = 10	Pre-treatment	Post-treatment	*p* value
**Tumor markers**			
PSA (ng/mL)	24.220 (3.507–88.354)	32.264 (2.245–164.363)	0.139
LDH (IU/L)	212 (172–281)	189 (152–320)	0.441
**Bone markers**			
ALP (IU/L)	355 (227–949)	276 (138–579)	0.066
BAP (IU/L)	30.3 (13.8–79.2)	14.4 (7.1–39.7)	0.066
ICTP (ng/mL)	8.4 (4.3–25.5)	11.9 (5.4–28.0)	0.260
**Imaging findings**			
EOD 1/2/3/4	1/1/3/5	1/2/4/3	0.129
BSI (%)	3.968 (0.588–7.430)	1.702 (0.316–6.876)	0.110
SUVmax	35.70 (17.06–55.03)	21.76 (14.24–37.68)	0.173
OMBV	1191.21 (93.80–1948.01)	512.97 (71.75–1697.02)	0.011
OTBU	9746.18 (1280.01– 17,587.11)	3151.47 (510.00– 13,172.21)	0.011

BSI = bone scan index; SUVmax = maximum standardized uptake value; OMBV = overall metabolic bone volume; OTBU = overall total bone uptake

**Table 3:** Change in parameters between pre- and post-treatment among patients with no response to Ra-223 therapy

N = 14	Pre-treatment	Post-treatment	*p* value
**Tumor markers**			
PSA (ng/mL)	39.370 (8.709–112.064)	93.173 (9.044–322.338)	0.011
LDH (IU/L)	215 (190–303)	272 (192–432)	0.039
**Bone markers**			
ALP (IU/L)	303 (188–428)	263 (187–677)	0.158
BAP (IU/L)	23.4 (8.0–39.1)	16.6 (8.1–54.5)	0.101
ICTP (ng/mL)	6.0 (4.7–11.1)	11.3 (5.1–19.2)	0.011
**Imaging findings**			
EOD 1/2/3/4	1/1/8/4	0/2/6/6	0.129
BSI (%)	1.943 (0.393–3.833)	2.359 (0.599–3.862)	0.003
SUVmax	28.62 (19.61–47.13)	24.11 (15.50–32.13)	0.006
OMBV	498.46 (294.53–829.67)	856.13 (410.80–1158.83)	0.002
OTBU	3569.10 (1852.86–7711.56)	6606.40 (2556.30– 10,244.55)	0.002

PSA = prostate-specific antigen; LDH = lactate dehydrogenase; ALP = alkaline phosphatase; BAP = bone-specific alkaline phosphatase; ICTP = type I collagen telopeptide; EOD = extent of disease; BSI = bone scan index; SUVmax = maximum standardized uptake value; OMBV = overall metabolic bone volume; OTBU = overall total bone uptake

**Fig. 2:**
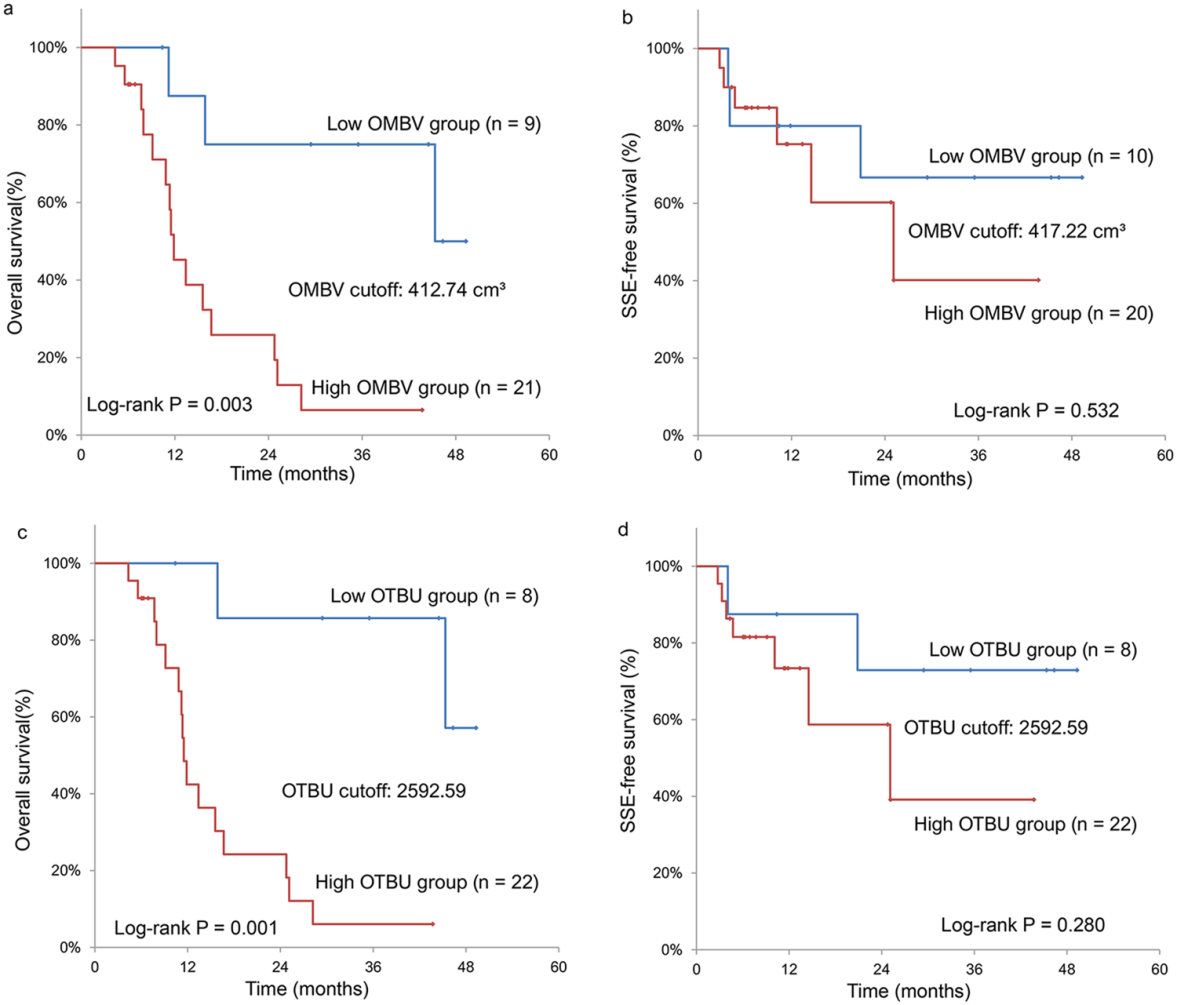
Kaplan-Meier curves for overall survival (OS) and time to symptomatic skeletal event (TTSE) stratified by OMBV and OTBU at baseline. Patients were dichotomized at ROC-derived Youden-index cut-offs (OS: OMBV 412.74 cm^3^, OTBU 2,592.59; TTSE: OMBV 417.22 cm^3^, OTBU 2,592.59). (**a**) OMBV vs OS (low *n* = 10, high *n* = 20; log-rank *p* = 0.003); (**b**) OMBV vs TTSE (low *n* = 11, high *n* = 19; log-rank *p* = 0.532); (**c**) OTBU vs OS (low *n* = 8, high *n* = 22; log-rank *p* = 0.001); (**d**) OTBU vs TTSE (low *n* = 8, high *n* = 22; log-rank *p* = 0.280). The y-axis represents the cumulative event-free rate. OS = overall survival; TTSE = time to symptomatic skeletal event; OMBV = overall metabolic bone volume; OTBU = overall total bone uptake

The median follow-up duration was 12 months (IQR 7–29). During the observation period, 18 of 30 patients (60%) died, and 9 patients (30%) experienced symptomatic skeletal events, including pathological fractures, spinal cord compression, or requirement for palliative radiation to bone. Kaplan-Meier analysis demonstrated significantly shorter OS in patients with high baseline OMBV and OTBU compared with those with low values (*p* < 0.05 by log-rank test; Fig. 2). The prognostic separation was particularly pronounced for OTBU: patients in the high OTBU group demonstrated a mortality rate of 73% compared with only 25% in the low OTBU group. Univariate Cox regression analysis for OS identified multiple significant prognostic factors (Table 4). For OS, time to CRPC and the number of prior CRPC treatment lines (≥3 vs < 3) did not reach statistical significance (*p* = 0.339 and *p* = 0.279, respectively). For PFS, the number of prior CRPC lines (≥3 vs < 3) showed an exploratory univariate association with a shorter interval to imaging-confirmed progression (HR 2.4648; 95% CI 1.0546–5.7608; *p* = 0.037; Table 5), consistent with the observation that heavily pretreated patients progressed earlier under Ra-223; this association is reported here as hypothesis-generating rather than confirmatory. Concomitant abiraterone exposure during Ra-223 showed a similar exploratory association with shorter PFS (HR 2.3210; *p* = 0.038), which should be interpreted cautiously given the non-randomized concomitant-therapy assignment and the likelihood of residual confounding by baseline tumor burden. For TTSE, the prior-line variable remained non-significant (*p* = 0.485; Table 6). Among imaging parameters, both OMBV (*p* < 0.001) and OTBU (*p* < 0.001) emerged as significant predictors, along with BSI (*p* = 0.001). Laboratory markers, including LDH (*p* = 0.001), ALP (*p* = 0.008), BAP (*p* = 0.009), and ICTP (*p* = 0.041), also demonstrated prognostic significance. Completion of the full Ra-223 course was strongly associated with improved OS (*p* < 0.001). In the secondary analyses, completion of the full Ra-223 course was again the strongest predictor of PFS (HR 0.1133, 95% CI 0.0412–0.3116; *p* < 0.001), along with prior number of CRPC lines ≥ 3 (*p* = 0.037), LDH (*p* = 0.019), ALP (*p* = 0.023), BAP (*p* = 0.041), and concomitant abiraterone (*p* = 0.038) (Table 5). Notably, baseline OMBV and OTBU did not reach statistical significance for PFS (*p* = 0.101 and 0.119, respectively), in contrast to their significant association with OS. For TTSE, only completion of the full Ra-223 course (HR 0.0658, 95% CI 0.0073–0.5941; *p* = 0.015) and LDH (*p* = 0.048) were significant predictors, while imaging parameters including OMBV, OTBU, BSI, and SUVmax showed no significant association (Table 6).

**Table 4:** Univariate Cox regression analysis for OS

Variable	Hazard Ratio	95% CI	*p* value
Age	1.0527	0.9816–1.1290	0.1498
Gleason score	0.7365	0.4511–1.2022	0.2212
Time to CRPC (months)	1.0072	0.9925–1.0222	0.3390
Prior number of CRPC lines ≥ 3 (PCWG3)	1.7261	0.6421–4.6401	0.2793
**Tumor markers at Ra-223**			
PSA	1.0001	0.9993–1.0010	0.7461
LDH	1.0055	1.0021–1.0090	0.0014
**Bone markers at Ra-223**			
ALP	1.0007	1.0002–1.0012	0.0079
BAP	1.0060	1.0015–1.0105	0.0091
ICTP	1.0521	1.0020–1.1046	0.0412
**Imaging findings at Ra-223**			
EOD	1.5344	0.8759–2.6876	0.1344
BSI	1.4146	1.1486–1.7422	0.0011
SUVmax	1.0138	0.9979–1.0301	0.0896
OMBV	1.0013	1.0006–1.0020	0.0006
OTBU	1.0001	1.0001–1.0002	0.0009
Dichotomized baseline OMBV (≥412.74 cm^3^)	6.0320	1.6880–21.5546	0.0006
Dichotomized baseline OTBU (≥2592.59)	9.4492	2.0968–42.5826	0.0009
**Ra-223 therapy**			
Completion of the injection	0.0571	0.0105–0.3106	0.0009
Positive response	0.7354	0.2704–2.0003	0.5472
Concomitant therapy during Ra-223			
Enzalutamide	0.6985	0.2634–1.8527	0.4710
Abiraterone	2.3163	0.8992–5.9666	0.0819
PSL	2.4077	0.7870–7.3664	0.1235
BMA	0.8357	0.3281–2.1289	0.7068

OS = overall survival; CI = confidence interval; CRPC = castration-resistant prostate cancer; PSA = prostate-specific antigen; LDH = lactate dehydrogenase; ALP = alkaline phosphatase; BAP = bone-specific alkaline phosphatase; ICTP = type I collagen telopeptide; EOD = extent of disease; BSI = bone scan index; SUVmax = maximum standardized uptake value; OMBV = overall metabolic bone volume; OTBU = overall total bone uptake; PSL = prednisolone; BMA = bone-modifying agent

**Table 5:** Univariate Cox regression analysis for PFS

Variable	Hazard Ratio	95% CI	p value
Age	1.0366	0.9800–1.0964	0.2098
Gleason score	0.9686	0.6174–1.5195	0.8895
Time to CRPC (months)	1.0048	0.9927–1.0170	0.4363
Prior number of CRPC lines ≥ 3 (PCWG3)	2.4648	1.0546–5.7608	0.0373
**Tumor markers at Ra-223**			
PSA	1.0021	0.9996–1.0046	0.0940
LDH	1.0034	1.0006–1.0062	0.0187
**Bone markers at Ra-223**			
ALP	1.0005	1.0001–1.0009	0.0233
BAP	1.0039	1.0002–1.0077	0.0408
ICTP	1.0027	0.9702–1.0362	0.8728
**Imaging findings at Ra-223**			
EOD	1.1529	0.7255–1.8320	0.5471
BSI	1.1383	0.9831–1.3179	0.0833
SUVmax	1.0095	0.9945–1.0247	0.2153
OMBV	1.0005	0.9999–1.0011	0.1005
OTBU	1.0000	1.0000–1.0001	0.1194
**Ra-223 therapy**			
Completion of the injection	0.1133	0.0412–0.3116	<0.0001
Positive response	0.6579	0.3054–1.4174	0.2850
Concomitant therapy during Ra-223			
Enzalutamide	0.4956	0.2248–1.0926	0.0818
Abiraterone	2.3210	1.0462–5.1490	0.0384
PSL	1.7313	0.7954–3.7684	0.1666
BMA	0.9354	0.4309–2.0308	0.8660

PFS = progression-free survival; CI = confidence interval; CRPC = castration-resistant prostate cancer; PSA = prostate-specific antigen; LDH = lactate dehydrogenase; ALP = alkaline phosphatase; BAP = bone-specific alkaline phosphatase; ICTP = type I collagen telopeptide; EOD = extent of disease; BSI = bone scan index; SUVmax = maximum standardized uptake value; OMBV = overall metabolic bone volume; OTBU = overall total bone uptake; PSL = prednisolone; BMA = bone-modifying agent

**Table 6:** Univariate Cox regression analysis for TTSE

Variable	Hazard Ratio	95% CI	p value
Age	1.0271	0.9332–1.1304	0.5848
Gleason score	0.7452	0.3621–1.5337	0.4245
Time to CRPC (months)	1.0113	0.9914–1.0315	0.2676
Prior number of CRPC lines ≥ 3 (PCWG3)	1.6459	0.4060–6.6721	0.4853
**Tumor markers at Ra-223**			
PSA	0.9984	0.9907–1.0062	0.6921
LDH	1.0039	1.0000–1.0078	0.0484
**Bone markers at Ra-223**			
ALP	0.9998	0.9985–1.0011	0.7655
BAP	0.9987	0.9870–1.0105	0.8226
ICTP	0.9187	0.7868–1.0727	0.2834
**Imaging findings at Ra-223**			
EOD	1.5545	0.6850–3.5275	0.2914
BSI	1.0229	0.7752–1.3497	0.8730
SUVmax	1.0096	0.9842–1.0358	0.4620
OMBV	1.0001	0.9990–1.0012	0.8539
OTBU	1.0000	0.9999–1.0001	0.8868
**Ra-223 therapy**			
Completion of the injection	0.0658	0.0073–0.5941	0.0154
Positive response	0.1482	0.0183–1.2002	0.0736
Concomitant therapy during Ra-223			
Enzalutamide	1.4977	0.3997–5.6114	0.5490
Abiraterone	0.8834	0.2184–3.5727	0.8620
PSL	1.2491	0.3069–5.0841	0.7561
BMA	0.3298	0.0811–1.3414	0.1212

TTSE = time to symptomatic skeletal event; CI = confidence interval; CRPC = castration-resistant prostate cancer; PSA = prostate-specific antigen; LDH = lactate dehydrogenase; ALP = alkaline phosphatase; BAP = bone-specific alkaline phosphatase; ICTP = type I collagen telopeptide; EOD = extent of disease; BSI = bone scan index; SUVmax = maximum standardized uptake value; OMBV = overall metabolic bone volume; OTBU = overall total bone uptake; PSL = prednisolone; BMA = bone-modifying agent

### Case presentations

**Fig. 3:**
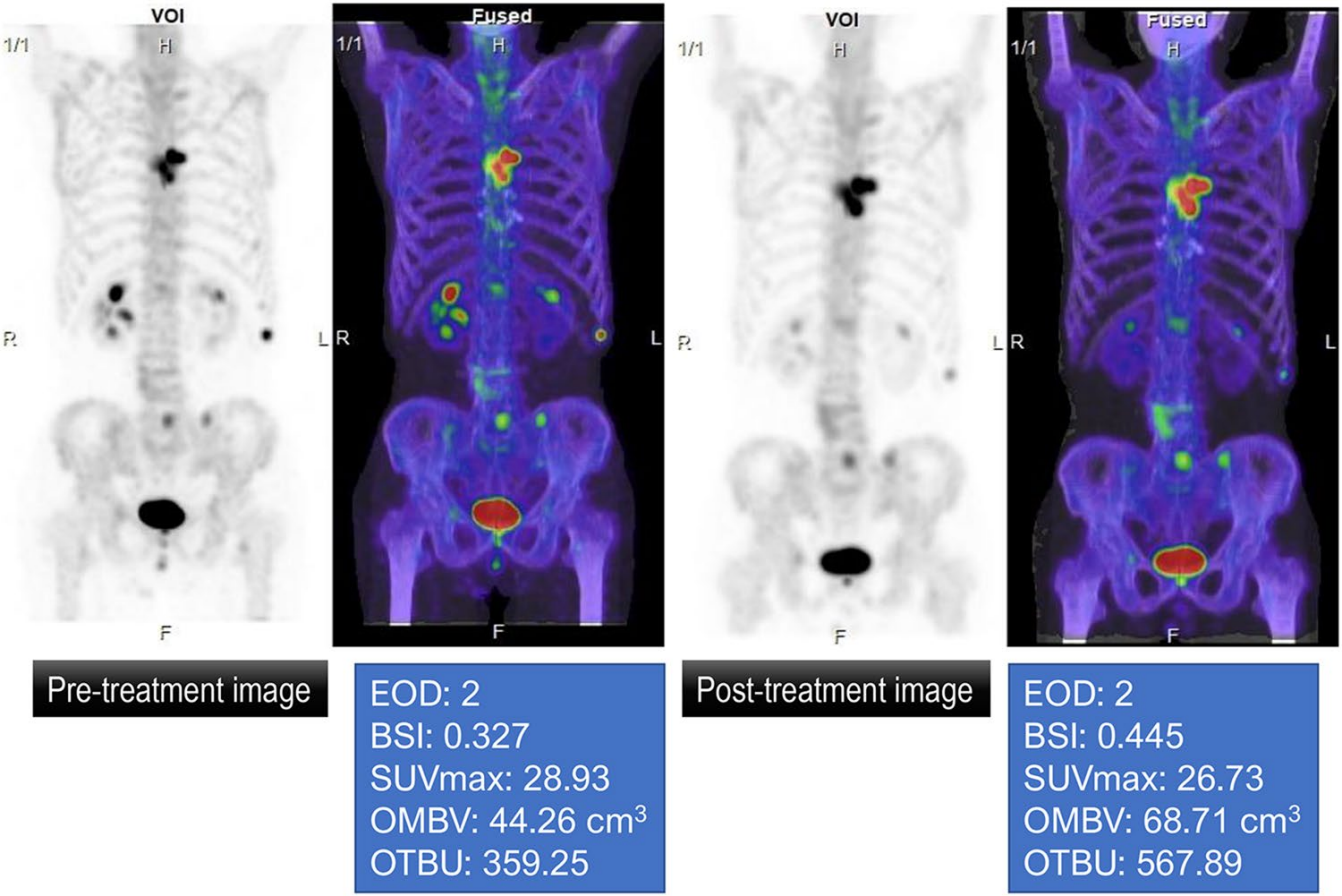
Representative case from the low OMBV and OTBU groups. Pre-treatment bone SPECT/CT of a 78-year-old man with CRPC showed multiple bone metastases (EOD = 2) in the 6th and 7th thoracic vertebrae, sacrum, and left ilium, with OMBV of 44.26 cm^3^ and OTBU of 359.25. Post-treatment bone SPECT/CT after the completion of Ra-223 therapy showed OMBV of 68.71 cm^3^ (+55.2%) and OTBU of 567.89 (+58.1%), indicating imaging-confirmed progression. Despite this post-treatment progression, the patient survived 883 days, illustrating that low baseline tumor burden can be associated with longer overall survival even when imaging parameters progress. CRPC = castration-resistant prostate cancer; EOD = extent of disease; OMBV = overall metabolic bone volume; OTBU = overall total bone uptake

Figure 3 shows a representative case from the low OTBU group. This 78-year-old man had a mildly elevated PSA of 5.322 ng/mL (normal upper limit: 4.0 ng/mL) and seven bone metastases (EOD = 2). The pre-treatment bone SPECT/CT revealed osteoblastic metastases with increased tracer uptake in the 6th and 7th thoracic vertebrae, the sacrum, and the left ilium. Quantitative analysis using GI-BONE yielded baseline values of SUVmax 28.93, OMBV 44.26 cm^3^, and OTBU 359.25. The patient completed the full Ra-223 course without treatment-limiting toxicities. Post-treatment imaging demonstrated increases in volumetric parameters (SUVmax 26.73, OMBV 68.71 cm^3^, OTBU 567.89), representing a 55.2% increase in OMBV (from 44.26 cm^3^) and a 58.1% increase in OTBU (from 359.25). Despite radiographic evidence of disease progression, this patient with low baseline OTBU survived 883 days (approximately 29 months) following initiation of Ra-223 therapy, exceeding the cohort median follow-up of 12 months, illustrating the favorable prognostic implications of low baseline tumor burden.

**Fig. 4:**
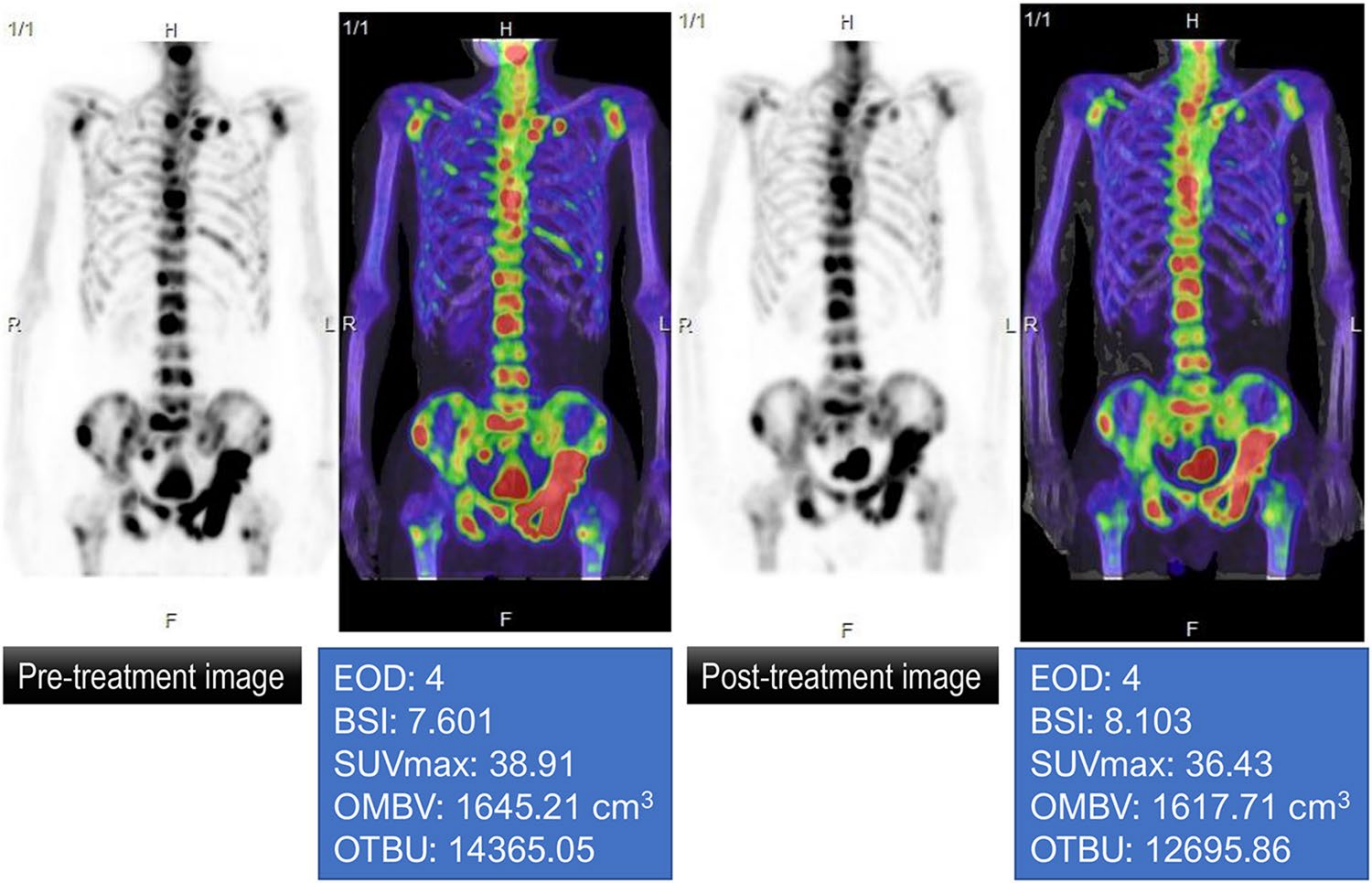
Case of an 82-year-old man with CRPC and bone metastases in the high OMBV and OTBU groups. Pre-treatment bone SPECT/CT showed extensive multiple bone metastases with no superscan (EOD = 4). Pre-treatment OMBV and OTBU were 1645.21 and 14,365.05, respectively. After the completion of the entire course of Ra-223, post-treatment bone SPECT/CT showed further extended diffuse bone metastases in the whole hematopoietic bone marrow region, with OMBV of 1617.71 and OTBU of 12,695.86. Despite minimal metabolic changes post-treatment, the patient died at 345 days, illustrating an adverse prognosis with a high baseline tumor burden

Figure 4 shows a representative case from the high OTBU group. This 82-year-old man showed a high PSA of 325.490 ng/mL and diffuse bone metastases involving the axial skeleton (vertebrae, pelvis, ribs, and sternum) and proximal appendicular skeleton without superscan (EOD = 4). Baseline quantitative parameters reflected substantial tumor burden: SUVmax 38.91, OMBV 1,645.21 cm^3^, and OTBU 14,365.05—values approximately 37-fold and 40-fold higher than those observed in the low OTBU case, respectively. Despite completion of the full Ra-223 course without treatment-limiting adverse events, post-treatment imaging revealed persistent diffuse metastatic involvement throughout the hematopoietic bone marrow. Post-treatment parameters (SUVmax 36.43, OMBV 1,617.71 cm^3^, OTBU 12,695.86) indicated only minimal decreases (OMBV: −1.7%, OTBU: −11.6%), reflecting inadequate therapeutic response. This patient experienced a fatal outcome 345 days after initiation of therapy, underscoring the adverse prognostic significance of high baseline skeletal tumor burden.

## Discussion

This study evaluated the prognostic value of volumetric parameters derived from quantitative bone SPECT/CT in CRPC patients undergoing Ra-223 therapy. Pre-treatment OTBU emerged as a significant prognostic marker for OS (*p* < 0.001), with OMBV also demonstrating prognostic value (*p* < 0.001). Patients with high baseline OTBU demonstrated 73% mortality rate compared with 25% in the low OTBU group. Serial quantitative assessment documented treatment-induced decreases in OMBV and OTBU among responders (averaging 57 and 68%), consistent with prior automated bone scan index studies demonstrating that treatment-associated reduction correlates with prolonged survival.

The therapeutic landscape for mCRPC has evolved substantially, with current options including ARSIs, taxane chemotherapy, bone-targeting agents, and radioligand therapies. Ra-223 is specifically recommended for patients with symptomatic bone-predominant disease without visceral metastases, and ^177^Lu-PSMA-617 has emerged as an alternative for PSMA-positive patients. Ra-223 is the only bone-targeting radionuclide demonstrating OS improvement in symptomatic mCRPC patients, making baseline assessment of bone metastatic extent critical for treatment planning. Previous studies using ^18^F-NaF PET/CT have shown that high baseline tumor burden indices (TLF10) are associated with shorter OS in Ra-223-treated patients (Etchebehere et al. 2015), and that pre-treatment ^18^F-fluoride and ^223^Ra imaging may predict lesion-level response to Ra-223 therapy (Murray et al. 2017). That high diphosphonate uptake, as measured by quantitative SPECT/CT, is an independent prognostic variable (Umeda et al. 2018). Our findings using OMBV and OTBU extend these observations by demonstrating that quantitative bone SPECT/CT provides comparable prognostic stratification using widely available technology.

SPECT/CT provides superior diagnostic accuracy compared with conventional planar bone scintigraphy through CT attenuation correction and additional anatomical information, enabling higher diagnostic confidence and inter-reviewer agreement. Our findings are consistent with Etchebehere et al., who demonstrated that volumetric parameters from ^18^F-NaF-PET/CT (TLF10 and FTV10) provided prognostic stratification in CRPC patients (Etchebehere et al. 2015), and Umeda et al., who reported that LUV and TLU derived from ^99m^Tc-diphosphonate SPECT/CT may provide higher prognostic value than conventional EOD or BSI (Umeda et al. 2018). Dittmann et al. further demonstrated that quantitative bone SPECT/CT parameters obtained before Ra-223 therapy provide prognostic information for OS in mCRPC (Dittmann et al. 2021). Our OMBV and OTBU parameters appear to demonstrate consistent prognostic performance comparable to these indices, suggesting that volumetric assessment of total metabolic bone metastasis burden may serve as a robust prognostic biomarker across different methodologies.

Our volumetric parameters complement established prognostic factors in mCRPC, including PSADT, performance status, baseline PSA, LDH, ALP, hemoglobin, and the presence of visceral metastases, by providing direct, imaging-based quantification of total skeletal tumor burden, potentially capturing disease characteristics not fully reflected in serum biomarkers alone. The prognostic performance of OMBV and OTBU can be attributed to multiple biological factors: mCRPC is bone-predominant, with approximately 90% of patients affected by skeletal lesions; bone marrow depletion by tumor expansion represents a significant cause of cancer-specific death, extensive skeletal involvement increases the likelihood that resistant clones exist within the heterogeneous metastatic population, and bone marrow infiltration compromises hematopoietic reserve, increasing susceptibility to treatment-limiting myelosuppression.

Our observation that patients in the high OTBU group were less likely to complete the full Ra-223 course (64% vs 100%, *p* = 0.045) is consistent with Dittmann et al., who reported that patients unable to complete Ra-223 treatment due to progression or cytopenia showed significantly higher baseline uptake on quantitative bone SPECT/CT (Dittmann et al. 2021). These findings suggest that quantitative volumetric assessment may help identify patients at risk for treatment-limiting toxicities, enabling proactive interventions, and that massive skeletal tumor burden may overwhelm the therapeutic capacity of standard Ra-223 dosing (Dittmann et al. 2021). Technically, three-dimensional volumetric assessment overcomes planar imaging limitations, including superimposition artifacts and incomplete disease quantification, enabling accurate lesion identification, individual lesion volume quantification, and summation across all skeletal sites with improved accuracy and reproducibility through CT-based attenuation correction.

The robust prognostic stratification achieved through pre-treatment OMBV and OTBU assessment has important clinical implications for objective risk stratification and treatment planning. Given ERA-223 findings demonstrating increased fracture risk with ARSI-Ra-223 combinations, high-risk patients may particularly benefit from mandatory bone-protective agents as confirmed by the PEACE-3 trial. The sensitivity of OMBV and OTBU for detecting therapeutic response provides objective imaging biomarkers for treatment monitoring, potentially guiding treatment intensification for high-volume disease. While quantitative ^18^F-NaF PET/CT has been considered promising for Ra-223 response assessment, our findings suggest that quantitative SPECT/CT may provide a more widely accessible alternative using standard clinical systems. The integration of these imaging biomarkers into clinical decision-making algorithms, potentially combined with molecular markers such as homologous recombination repair (HRR) gene alterations, which are present in 20–30% of CRPC tumors, could enable more comprehensive risk assessment and precise treatment planning in the evolving mCRPC landscape.

An important observation from our endpoint-specific analyses is that baseline OMBV and OTBU were significantly associated with OS but not with PFS or TTSE. Several biological and methodological considerations may explain this pattern. Volumetric tumor burden parameters capture the total metabolic mass of skeletal disease, which plausibly reflects systemic disease severity and hematopoietic reserve more directly than the timing of individual progression events or skeletal-related complications. OS integrates the cumulative impact of disease burden, treatment tolerability, and competing risks, whereas PFS and TTSE depend on the timing of discrete events that may be influenced by local lesion biology, symptom threshold, and clinical surveillance intensity. In addition, with only 18 deaths and 9 symptomatic skeletal events in 30 patients, statistical power to detect imaging-based associations is inherently lower for TTSE than for OS; for PFS, although all 30 patients had imaging-confirmed progression (no censoring), the modest sample size likewise limits power to detect continuous OMBV/OTBU effects; the non-significant trends for OMBV (*p* = 0.101) and OTBU (*p* = 0.119) in the PFS analysis are compatible with a true but under-powered association. Furthermore, completion of the full Ra-223 course was the dominant predictor across all three endpoints, and in the high-burden subgroup early discontinuation may attenuate the apparent prognostic signal of baseline volumetric parameters for progression-based endpoints while preserving their association with OS, which is less sensitive to short-term treatment exposure. These observations suggest that OMBV and OTBU are best positioned as global prognostic markers for overall outcome rather than as surrogates for short-term disease control under Ra-223 therapy, and larger cohorts will be needed to determine whether their association with PFS and TTSE reaches statistical significance.

Several limitations warrant acknowledgment. The small sample size (30 patients) constrains statistical power and precludes robust multivariable analysis. The univariate Cox analyses in Tables 4–6 included 20 candidate variables across three endpoints, totaling 60 univariate tests, and we did not adjust for multiple comparisons; borderline *p* values (approximately 0.03–0.05) should therefore be interpreted as hypothesis-generating rather than as confirmatory evidence. The retrospective single-center design introduces potential selection bias. Our cohort demonstrated a high prevalence of advanced disease (83% Gleason score 8–10, 87% EOD 3–4), which may reflect referral bias. The SUV threshold of 4.50 for lesion identification, while grounded in normal-bone reference values reported in prior literature, may still require site-specific calibration across different scanner platforms and reconstruction protocols. In addition, because the ROC-derived OMBV and OTBU cut-offs were both derived and tested within the same single-center cohort, resubstitution bias likely inflates the apparent magnitude of the dichotomized hazard ratios; external validation in an independent cohort is required before these thresholds can be adopted in clinical practice. Post-treatment imaging was not available for all patients (80%), potentially introducing attrition bias. Imaging-based PFS ascertainment depended on institutional imaging schedules and on post-transfer follow-up records, so patients with more frequent imaging may have had progression documented sooner; this introduces a potential follow-up-intensity bias that could not be fully eliminated in a retrospective cohort and may affect the PFS hazard ratios more than the OS or TTSE estimates. A further important limitation is the potential confounding effect of concomitant systemic therapies, particularly ARSIs. In our cohort, 22 of 30 patients (73%) continued enzalutamide or abiraterone during Ra-223 administration, and 6 additional patients had non-concomitant ARSI exposure before or after Ra-223. Because ARSIs demonstrate independent efficacy in mCRPC, the observed survival signals associated with baseline OMBV and OTBU cannot be fully attributed to Ra-223 alone; the exploratory association between concomitant abiraterone and shorter PFS (HR 2.3210; *p* = 0.038; Table 5) most plausibly reflects channeling of higher-burden or more heavily pretreated patients toward ARSI continuation rather than a causal detrimental effect, and should not be interpreted as evidence against combining the two agents. The retrospective nature of the dataset also precluded precise temporal reconstruction of all prior systemic agents. Despite these limitations, our findings provide preliminary evidence and may establish a foundation for larger multicenter validation studies.

Future investigations should pursue multicenter prospective validation with standardized imaging protocols to establish optimal SUV thresholds and confirm generalizability across different scanner platforms. The methodology may extend beyond Ra-223 to other bone-targeting radioligand therapies, including ^177^Lu-PSMA-617, where baseline tumor burden assessment is equally important for patient selection and response prediction. Integration of volumetric bone parameters with molecular profiling, including homologous recombination repair (HRR) gene alterations and PSMA expression status, could enable more comprehensive risk stratification algorithms. Artificial intelligence and machine learning approaches may facilitate automated lesion detection and volumetric quantification, enhancing reproducibility and clinical workflow efficiency.

## Conclusion

Quantitative bone SPECT/CT-derived OMBV and OTBU may serve as prognostic biomarkers in mCRPC patients treated with Ra-223, complementing established clinical and biochemical markers. Because the methodology is based on widely available SPECT/CT technology, these volumetric parameters may be integrable into routine clinical workflows, pending further validation. Prospective validation in larger multicenter cohorts is needed to confirm these findings.

## Data Availability

The datasets generated during the current study are available from the corresponding author on reasonable request.

## References

[CR1] Armstrong AJ, Garrett-Mayer E, de Wit R, Tannock I, Eisenberger M (2010) Prediction of survival following first-line chemotherapy in men with castration-resistant metastatic prostate cancer. Clin Cancer Res 16(1):203–211. 10.1158/1078-0432.CCR-09-2514 20008841 10.1158/1078-0432.CCR-09-2514

[CR2] Bodei L, Lam M, Chiesa C, Flux G, Brans B, Chiti A, Giammarile F (2008) EANM procedure guideline for treatment of refractory metastatic bone pain. Eur J Nucl Med Mol Imag 35(10):1934–1940. 10.1007/s00259-008-0841-y 10.1007/s00259-008-0841-y 18649080

[CR3] Brown JE, Cook RJ, Major P, Lipton A, Saad F, Smith M, Lee K-A, Zheng M, Hei Y-J, Coleman RE (2005) Bone turnover markers as predictors of skeletal complications in prostate cancer, lung cancer, and other solid tumors. JNCI J The Natl Cancer Inst 97(1):59–69. 10.1093/jnci/dji002 10.1093/jnci/dji002 15632381

[CR4] Bruland OS, Nilsson S, Fisher DR, Larsen RH (2006) High-linear energy transfer irradiation targeted to skeletal metastases by the alpha emitter 223Ra: adjuvant or alternative to conventional modalities? Clin Cancer Res 12(20):6250s–6257s. 10.1158/1078-0432.CCR-06-0841 10.1158/1078-0432.CCR-06-0841 17062709

[CR5] Bubendorf L, Schopfer A, Wagner U, Sauter G, Moch H, Willi N, Gasser TC, Mihatsch MJ (2000) Metastatic patterns of prostate cancer: an autopsy study of 1, 589 patients. Hum Pathol 31(5):578–583. 10.1053/hp.2000.6698 10836297 10.1053/hp.2000.6698

[CR6] Cook GJR, Azad G, Padhani AR (2016) Bone imaging in prostate cancer: the evolving roles of nuclear medicine and radiology. Clin Transl Imag 4(6):439–447. 10.1007/s40336-016-0196-5 10.1007/s40336-016-0196-5 PMC5118401 27933280

[CR7] Cook RJ, Coleman R, Brown J, Lipton A, Major P, Hei YJ, Saad F, Smith MR (2006) Markers of bone metabolism and survival in men with hormone-refractory metastatic prostate cancer. Clin Cancer Res 12(11):3361–3367. 10.1158/1078-0432.CCR-06-0269 16740758 10.1158/1078-0432.CCR-06-0269

[CR8] de Bono Js, Logothetis CJ, Molina A, de Bono JS, Fizazi K, North S, Chu L, Chi KN, Jones RJ, Goodman OB, Saad F, Staffurth JN, Mainwaring P, Harland S, Flaig TW, Hutson TE, Cheng T, Patterson H, Hainsworth JD, Ryan CJ, Sternberg CN, Ellard SL, Fléchon A, Saleh M, Scholz M, Efstathiou E, Zivi A, Bianchini D, Loriot Y, Chieffo N, Kheoh T, Haqq CM, Scher HI (2011) Abiraterone and increased survival in metastatic prostate cancer. N Engl J Med 364(21):1995–2005. 10.1056/NEJMoa1014618 21612468 10.1056/NEJMoa1014618 PMC3471149

[CR9] Dennis ER, Jia X, Mezheritskiy IS, Stephenson RD, Schoder H, Fox JJ, Heller G, Scher HI, Larson SM, Morris MJ (2012) Bone scan index: a quantitative treatment response biomarker for castration-resistant metastatic prostate cancer. JCO 30(5):519–524. 10.1200/JCO.2011.36.5791 10.1200/JCO.2011.36.5791 PMC3295554 22231045

[CR10] Dittmann H, Kaltenbach S, Weissinger M, Fiz F, Martus P, Pritzkow M, Kupferschlaeger J, la Fougère C (2021) The prognostic value of quantitative bone SPECT/CT before 223 Ra treatment in metastatic castration-resistant prostate cancer. J Nucl Med 62(1):48–54. 10.2967/jnumed.119.240408 32444369 10.2967/jnumed.119.240408

[CR11] Etchebehere EC, Araujo JC, Fox PS, Swanston NM, Macapinlac HA, Rohren EM (2015) Prognostic factors in patients treated with 2 2 3 Ra: the role of skeletal tumor burden on baseline 18F-fluoride PET/CT in predicting overall survival. J Nucl Med 56(8):1177–1184. 10.2967/jnumed.115.158626 26069307 10.2967/jnumed.115.158626

[CR12] Even-Sapir E, Metser U, Mishani E, Lievshitz G, Lerman H, Leibovitch I (2006) The detection of bone metastases in patients with high-risk prostate cancer: 99mTc-MDP planar bone scintigraphy, single- and multi-field-of-view SPECT, 18F-fluoride PET, and 18F-fluoride PET/CT. J Nucl Med 47(2):287–297 16455635

[CR13] Fizazi K, Carducci M, Smith M, Damião R, Brown J, Karsh L, Milecki P, Shore N, Rader M, Wang H, Jiang Q, Tadros S, Dansey R, Goessl C (2011) Denosumab versus zoledronic acid for treatment of bone metastases in men with castration-resistant prostate cancer: a randomised, double-blind study. Lancet 377(9768):813–822. 10.1016/S0140-6736(10)62344-6 21353695 10.1016/S0140-6736(10)62344-6 PMC3090685

[CR14] Fizazi K, Massard C, Smith M, Rader M, Brown J, Milecki P, Shore N, Oudard S, Karsh L, Carducci M, Damião R, Wang H, Ying W, Goessl C (2015) Bone-related parameters are the main prognostic factors for overall survival in men with bone metastases from castration-resistant prostate cancer. Eur Urology 68(1):42–50. 10.1016/j.eururo.2014.10.001 10.1016/j.eururo.2014.10.001 25449207

[CR15] Halabi S, Lin CY, Kelly WK, Fizazi KS, Moul JW, Kaplan EB, Morris MJ, Small EJ (2014) Updated prognostic model for predicting overall survival in first-line chemotherapy for patients with metastatic castration-resistant prostate cancer. JCO 32(7):671–677. 10.1200/JCO.2013.52.3696 10.1200/JCO.2013.52.3696 PMC3927736 24449231

[CR16] Hou X, He Y, Liu G, Chen S, Shi H et al (2024) SPECT/CT imaging: quantifying 99mTc-MDP concentration in the spine and pelvis. Ann Nucl Med 38(12):933–942. 10.1007/s12149-024-01967-9 39154304 10.1007/s12149-024-01967-9

[CR17] Imbriaco M, Larson SM, Yeung HW, Mawlawi OR, Erdi Y, Venkatraman ES, Scher HI (1998) A new parameter for measuring metastatic bone involvement by prostate cancer: the bone scan index. Clin Cancer Res 4(7):1765–1772 9676853

[CR18] Jorgensen T, Muller C, Kaalhus O, Danielsen HE, Tveter KJ (1995) Extent of disease based on initial bone scan: important prognostic predictor for patients with metastatic prostatic cancer. Eur Urol 28(1):40–46. 10.1159/000475018 8521893 10.1159/000475018

[CR19] Kaboteh R, Damber JE, Gjertsson P, Höglund P, Lomsky M, Ohlsson M, Edenbrandt L (2013) Bone scan index: a prognostic imaging biomarker for high-risk prostate cancer patients receiving primary hormonal therapy. EJNMMI Res 3(1):9. 10.1186/2191-219X-3-9 23384286 10.1186/2191-219X-3-9 PMC3570487

[CR20] Kuji I, Yamane T, Seto A, Yasumizu Y, Shirotake S, Oyama M (2017) Skeletal standardized uptake values obtained by quantitative SPECT/CT as an osteoblastic biomarker for the discrimination of active bone metastasis in prostate cancer. Eur J Hybrid Imag 1(1):2. 10.1186/s41824-017-0006-y 10.1186/s41824-017-0006-y PMC5954671 29782587

[CR21] Lange PH, Vessella RL (1998) Mechanisms, hypotheses and questions regarding prostate cancer micrometastases to bone. Cancer Metastasis Rev 17(4):331–336. 10.1023/A:1006106209527 10453276 10.1023/a:1006106209527

[CR22] Logothetis CJ, Lin SH (2005) Osteoblasts in prostate cancer metastasis to bone. Nat Rev Cancer 5(1):21–28. 10.1038/nrc1528 15630412 10.1038/nrc1528

[CR23] Miyoshi Y, Yoneyama S, Kawahara T, Hattori Y, Teranishi J-I, Kondo K, Moriyama M, Takebayashi S, Yokomizo Y, Yao M, Uemura H, Noguchi K (2016) Prognostic value of the bone scan index using a computer-aided diagnosis system for bone scans in hormone-naive prostate cancer patients with bone metastases. BMC Cancer 16(1):128. 10.1186/s12885-016-2176-6 26896160 10.1186/s12885-016-2176-6 PMC4759962

[CR24] Moridera K, Kitajima K, Yoshikawa K, Takaoka K, Tsuchitani T, Noguchi K, Kishimoto H, Yamakado K (2022) Usefulness of quantitative bone SPECT/CT for medication-related osteonecrosis of the jaw in clinical settings. Jpn J Radiol 40(5):492–499. 10.1007/s11604-021-01226-1 34851501 10.1007/s11604-021-01226-1

[CR25] Murray I, Chittenden SJ, Denis-Bacelar AM, Hindorf C, Parker CC, Chua S, Flux GD (2017) The potential of 2 2 3 Ra and 1 8 F-fluoride imaging to predict bone lesion response to treatment with 2 2 3 Ra-dichloride in castration-resistant prostate cancer. Eur J Nucl Med Mol Imag 44(11):1832–1844. 10.1007/s00259-017-3744-y 10.1007/s00259-017-3744-y PMC6175045 28612079

[CR26] Parker C, Nilsson S, Heinrich D, Helle SI, O’Sullivan JM, Fosså SD, Chodacki A, Wiechno P, Logue J, Seke M, Widmark A, Johannessen DC, Hoskin P, Bottomley D, James ND, Solberg A, Syndikus I, Kliment J, Wedel S, Boehmer S, Dall’Oglio M, Franzén L, Coleman R, Vogelzang NJ, O’Bryan-Tear CG, Staudacher K, Garcia-Vargas J, Shan M, Bruland OS, Sartor O (2013) Alpha emitter radium-223 and survival in metastatic prostate cancer. N Engl J Med 369(3):213–223. 10.1056/NEJMoa1213755 23863050 10.1056/NEJMoa1213755

[CR27] Patrick DL, Cleeland CS, von Moos R, Fallowfield L, Wei R, Öhrling K, Qian Y (2015) Pain outcomes in patients with bone metastases from advanced cancer: assessment and management with bone-targeting agents. Support Care Cancer 23(4):1157–1168. 10.1007/s00520-014-2525-4 25533578 10.1007/s00520-014-2525-4

[CR28] Rohani MFM, Yonan SNM, Tagiling N, Zainon MSF, Udin Y, Nawi NM et al (2020) Standardized uptake value from semiquantitative bone single-photon emission computed tomography/computed tomography in normal thoracic and lumbar vertebrae of breast cancer patients. Asian Spine J 14(5):629–638. 10.31616/asj.2019.0308 32213791 10.31616/asj.2019.0308 PMC7595810

[CR29] Roodman GD (2004) Mechanisms of bone metastasis. N Engl J Med 350(16):1655–1664. 10.1056/NEJMra030831 15084698 10.1056/NEJMra030831

[CR30] Ryan CJ, Smith MR, de Bono JS, Molina A, Logothetis CJ, de Souza P, Fizazi K, Mainwaring P, Piulats JM, Ng S, Carles J, Mulders PFA, Basch E, Small EJ, Saad F, Schrijvers D, Van Poppel H, Mukherjee SD, Suttmann H, Gerritsen WR, Flaig TW, George DJ, Yu EY, Efstathiou E, Pantuck A, Winquist E, Higano CS, Taplin M-E, Park Y, Kheoh T, Griffin T, Scher HI, Rathkopf DE (2013) Abiraterone in metastatic prostate cancer without previous chemotherapy. N Engl J Med 368(2):138–148. 10.1056/NEJMoa1209096 23228172 10.1056/NEJMoa1209096 PMC3683570

[CR31] Rybak LD, Rosenthal DI (2001) Radiological imaging for the diagnosis of bone metastases. Q J Nucl Med 45(1):53–64 11456376

[CR32] Saad F, Chen YM, Gleason DM, Chin J (2007) Continuing benefit of zoledronic acid in preventing skeletal complications in patients with bone metastases. Clin Genitourin Cancer 5(6):390–396. 10.3816/CGC.2007.n.022 17956712 10.3816/CGC.2007.n.022

[CR33] Saad F, Gleason DM, Murray R, Tchekmedyian S, Venner P, Lacombe L, Chin JL, Vinholes JJ, Goas JA, Zheng M (2004) Long-term efficacy of zoledronic acid for the prevention of skeletal complications in patients with metastatic hormone-refractory prostate cancer. JNCI J The Natl Cancer Inst 96(11):879–882. 10.1093/jnci/djh141 10.1093/jnci/djh141 15173273

[CR34] Saad F, Lipton A, Cook R, Chen YM, Smith M, Coleman R (2007) Pathologic fractures correlate with reduced survival in patients with malignant bone disease. Cancer 110(8):1860–1867. 10.1002/cncr.22991 17763372 10.1002/cncr.22991

[CR35] Sartor O, de Bono J, Chi KN, Fizazi K, Herrmann K, Rahbar K, Tagawa ST, Nordquist LT, Vaishampayan N, El-Haddad G, Park CH, Beer TM, Armour A, Pérez-Contreras WJ, DeSilvio M, Kpamegan E, Gericke G, Messmann RA, Morris MJ, Krause BJ (2021) Lutetium-177–PSMA-617 for Metastatic Castration-Resistant Prostate Cancer. N Engl J Med 385(12):1091–1103. 10.1056/NEJMoa2107322 34161051 10.1056/NEJMoa2107322 PMC8446332

[CR36] Scher HI, Fizazi K, Saad F, Taplin M-E, Sternberg CN, Miller K, de Wit R, Mulders P, Chi KN, Shore ND, Armstrong AJ, Flaig TW, Fléchon A, Mainwaring P, Fleming M, Hainsworth JD, Hirmand M, Selby B, Seely L, de Bono JS (2012) Increased survival with enzalutamide in prostate cancer after chemotherapy. N Engl J Med 367(13):1187–1197. 10.1056/NEJMoa1207506 22894553 10.1056/NEJMoa1207506

[CR37] Scher HI, Morris MJ, Stadler WM, Higano C, Basch E, Fizazi K, Antonarakis ES, Beer TM, Carducci MA, Chi KN, Corn PG, de Bono JS, Dreicer R, George DJ, Heath EI, Hussain M, Kelly WK, Liu G, Logothetis C, Nanus D, Stein MN, Rathkopf DE, Slovin SF, Ryan CJ, Sartor O, Small EJ, Smith MR, Sternberg CN, Taplin M-E, Wilding G, Nelson PS, Schwartz LH, Halabi S, Kantoff PW, Armstrong AJ (2016) Trial design and objectives for castration-resistant prostate cancer: updated recommendations from the prostate cancer clinical trials Working group 3. JCO 34(12):1402–1418. 10.1200/JCO.2015.64.2702 10.1200/JCO.2015.64.2702 PMC4872347 26903579

[CR38] Soloway MS, Hardeman SW, Hickey D, Todd B, Soloway S, Raymond J, Moinuddin M (1988) Stratification of patients with metastatic prostate cancer based on extent of disease on initial bone scan. Cancer 61(1):195–202. 10.1002/1097-0142(19880101)61:1<195::AID-CNCR2820610133>3.0.CO;2-Y 10.1002/1097-0142(19880101)61:1<195::aid-cncr2820610133>3.0.co;2-y 3334948

[CR39] Ulmert D, Kaboteh R, Fox JJ, Savage C, Evans MJ, Lilja H, Abrahamsson P-A, Björk T, Gerdtsson A, Bjartell A, Gjertsson P, Höglund P, Lomsky M, Ohlsson M, Richter J, Sadik M, Morris MJ, Scher HI, Sjöstrand K, Yu A, Suurküla M, Edenbrandt L, Larson SM (2012) A novel automated platform for quantifying the extent of skeletal tumour involvement in prostate cancer patients using the bone scan index. Eur Urology 62(1):78–84. 10.1016/j.eururo.2012.01.037 10.1016/j.eururo.2012.01.037 PMC3402084 22306323

[CR40] Umeda T, Koizumi M, Fukai S, Miyaji N, Motegi K, Nakazawa S, Takiguchi T (2018) Evaluation of bone metastatic burden by bone SPECT/CT in metastatic prostate cancer patients: defining threshold value for total bone uptake and assessment in radium-223 treated patients. Ann Nucl Med 32(2):105–113. 10.1007/s12149-017-1224-x 29243019 10.1007/s12149-017-1224-x PMC5797193

[CR41] Utsunomiya D, Shiraishi S, Imuta M, Tomiguchi S, Kawanaka K, Morishita S, Awai K, Yamashita Y (2006) Added value of SPECT/CT fusion in assessing suspected bone metastasis: comparison with scintigraphy alone and nonfused scintigraphy and CT. Radiology 238(1):264–271. 10.1148/radiol.2373041358 16304081 10.1148/radiol.2373041358

[CR42] Weinfurt KP, Castel LD, Li Y, Timbie JW, Glendenning GA, Schulman KA (2004) Health-related quality of life among patients with breast cancer receiving zoledronic acid or pamidronate disodium for metastatic bone lesions. Med Care 42(2):164–175. 10.1097/01.mlr.0000108746.69256.45 14734954 10.1097/01.mlr.0000108746.69256.45

[CR43] Weinfurt KP, Li Y, Castel LD, Saad F, Timbie JW, Glendenning GA, Schulman KA (2005) The significance of skeletal-related events for the health-related quality of life of patients with metastatic prostate cancer. Ann Oncol 16(4):579–584. 10.1093/annonc/mdi122 15734776 10.1093/annonc/mdi122

[CR44] Yamane T, Fukushima K, Shirotake S, Nishimoto K, Okabe T, Oyama M, Seto A, Kuji I (2021) Test–retest repeatability of quantitative bone SPECT/CT. Ann Nucl Med 35(3):338–346. 10.1007/s12149-020-01568-2 33400148 10.1007/s12149-020-01568-2

[CR45] Yap BK, Choo R, Deboer G, Klotz L, Danjoux C, Morton G (2003) Are serial bone scans useful for the follow-up of clinically localized, low to intermediate grade prostate cancer managed with watchful observation alone? BJU Int 91(7):613–617. 10.1046/j.1464-410X.2003.04169.x 12699470 10.1046/j.1464-410x.2003.04169.x

